# Domestic violence in Indian women: lessons from nearly 20 years of surveillance

**DOI:** 10.1186/s12905-022-01703-3

**Published:** 2022-04-21

**Authors:** Rakhi Dandona, Aradhita Gupta, Sibin George, Somy Kishan, G. Anil Kumar

**Affiliations:** 1 Public Health Foundation of India, Plot No. 47, Sector 44, Institutional Area, Gurugram, Haryana 122002 India; 2 Institute for Health Metrics and Evaluation, University of Washington, Seattle, USA

**Keywords:** Domestic violence, India, Intimate partner, Dowry, Suicide

## Abstract

**Background:**

Prevalence of self-reported domestic violence against women in India is high. This paper investigates the national and sub-national trends in domestic violence in India to prioritise prevention activities and to highlight the limitations to data quality for surveillance in India.

**Methods:**

Data were extracted from annual reports of National Crimes Record Bureau (NCRB) under four domestic violence crime-headings—cruelty by husband or his relatives, dowry death, abetment to suicide, and protection of women against domestic violence act. Rate for each crime is reported per 100,000 women aged 15–49 years, for India and its states from 2001 to 2018. Data on persons arrested and legal status of the cases were extracted.

**Results:**

Rate of reported cases of cruelty by husband or relatives in India was 28.3 (95% CI 28.1–28.5) in 2018, an increase of 53% from 2001. State-level variations in this rate ranged from 0.5 (95% CI  − 0.05 to 1.5) to 113.7 (95% CI 111.6–115.8) in 2018. Rate of reported dowry deaths and abetment to suicide was 2.0 (95% CI 2.0–2.0) and 1.4 (95% CI 1.4–1.4) in 2018 for India, respectively. Overall, a few states accounted for the temporal variation in these rates, with the reporting stagnant in most states over these years. The NCRB reporting system resulted in underreporting for certain crime-headings. The mean number of people arrested for these crimes had decreased over the period. Only 6.8% of the cases completed trials, with offenders convicted only in 15.5% cases in 2018. The NCRB data are available in heavily tabulated format with limited usage for intervention planning. The non-availability of individual level data in public domain limits exploration of patterns in domestic violence that could better inform policy actions to address domestic violence.

**Conclusions:**

Urgent actions are needed to improve the robustness of NCRB data and the range of information available on domestic violence cases to utilise these data to effectively address domestic violence against women in India.

**Supplementary Information:**

The online version contains supplementary material available at 10.1186/s12905-022-01703-3.

## Background

The Sustainable Development Goal (SDG) target 5 is to eliminate all forms of violence against women and girls, and the two indicators of progress towards this are the rates of intimate partner violence (IPV) and non-partner violence [1]. The World Health Organization (WHO) estimated a 26% prevalence of IPV in ever-married/partnered women aged 15 years or more globally in 2018, and this prevalence is higher at 35% for southern Asia region in which India falls [2]. The self-reported domestic violence (majority by an intimate partner) in any form is reported between 33 to 41% among ever-married women from India [3–8]. Furthermore, the suicide death rate among women in India was reported to be twice the global rate [9], and housewives account for the majority of suicide deaths, the reasons for which are documented as “personal/social” [10].

Domestic violence was first recognized as a punishable offence in India in 2005 with the passing of the Protection of Women from Domestic Violence Act (PWDVA) [11, 12]. A significant focus of domestic violence against women in India has been on dowry-related harassment. Dowry is the transfer of goods, money and/or property from the bride’s family to the groom or his family at the time of marriage [13], initially meant to provide funds to women who were unable to inherit family property [14]. Dowry is very prevalent in India [15], and it has propagated domestic violence as means to extract money or property from the bride and her family [13, 16]. While earlier sections of the Indian Penal Code (IPC) criminalized only dowry-related domestic violence, PWDVA expanded legal recourse for domestic violence beyond dowry harassment for more effective protection of the rights of women guaranteed under the Constitution who are victims of violence of any kind occurring within the family [11].

The major official source of surveillance for domestic violence in India are the reports compiled by the National Crime Records Bureau (NCRB) [17]. Though under-reporting in NCRB reports is well documented for certain types of injuries [9, 10, 18], it remains the most comprehensive longitudinal source of domestic violence available at the state-level for India. We undertook a situational analysis for the years 2001 to 2018 using the NCRB reports to highlight the trends in the reported magnitude of domestic violence over time, to highlight the variations within country that could facilitate prioritization of immediate actions for prevention, and to discuss the limitations of the available NCRB reports for surveillance.

## Methods

The primary source of the NCRB data is the First Information Report (FIR) completed by a police officer for any domestic violence incident which is compiled at the state level and provided to NCRB. FIR is a document prepared by police when they receive information about the commission of a cognizable offence either by the victim of the cognizable offence or by someone on their behalf [19, 20]. It captures the date, time and location of offence, the details of offence, the details of victim and person reporting the offence, and steps taken by the police after receiving these details. The NCRB reports provide summary data based on these FIRs, which we utilized from 2001 to 2018 available in the public domain for this analysis. The details of data extracted and utilized are described below.

### Type of data

Four crime headings corresponding to domestic violence related crimes against women were considered after consultation with legal experts who dealt with domestic violence cases based on the crime headings under which these are registered in India —cruelty by husband or his relatives, dowry death, abetment of suicide of women, and cases registered under PWDVA (Additional file 1: Table S1). A case is filed under ‘cruelty by husband or his relatives’ (Section 498A of the IPC) when there is evidence of violence causing grave injury or of harassment to fulfil an unlawful demand for property [21]. Case of death of a woman within 7 years of marriage with evidence of dowry harassment is filed under dowry death (IPC Section 304B) [22]. As domestic violence is known as a risk factor for death by suicide among married women, we also considered the cases registered under abetment of suicide of women [23]. The cases under the PWDVA act criminalize perpetrators of domestic violence, defined to include physical, verbal, sexual, emotional and economic abuse in addition to dowry-related violence [11]. The NCRB reports data based on the “Principal Offence Rule,” which means that regardless of the number of offences under which a case of domestic violence is legally registered, it is reported only under the most serious crime heading by the NCRB [24].

### Data extraction

NCRB reports included the number of cases filed as well as the number of victims under each of the four crime headings for 2014–2018 but reported only the number of cases filed from 2001 to 2013. The ratio of the number of cases to victims was 1.0 for 2014 to 2018, and hence we use the number of cases filed for this analysis from 2001 to 2018. Individual level-data is not published in the NCRB report.

Data for cruelty by husband or his relatives and for dowry death were available from 2001 to 2018, while data for abetment of suicide of women and PWDVA were available only from 2014 to 2018. We extracted the number of cases filed under each of the four domestic violence crime heads for each year for each state and for India. We also extracted data on the number of persons arrested under each crime category, which were available from 2001 to 2015 for the states and until 2018 for India. Here too, the data on abetment of suicide and PWDVA was available from 2014 to 2018 only. Lastly, we extracted data on the number of legal cases filed for these crimes and their current status in the judicial system. This legal data was available cumulatively for only India, and since it could not be extrapolated for each year from the tables, we analyzed this only for 2018.

### Data analysis

Our analysis was aimed at understanding trends in the rate for each type of domestic violence crime heading. We calculated the rate of cruelty by husband or his relatives and dowry deaths from 2001 to 2018, and the rate of abetment of suicide of women and PWDVA from 2014 to 2018. As the NCRB reports do not specify the age of women who had reported these crimes, we assumed the age group of women to be 15–49 years to estimate the rates as the previous reports on domestic violence in India are predominately for women aged 15–49 years [25–31]. We used the Global Burden of Disease (GBD) study 2019 state-wise annual population estimates for women aged 15–49 years as the denominator [32], and report the rates per 100,000 women aged 15–49 years with 95% confidence intervals (CI) estimated for these rates. We report these rates across three administrative splits: nationally, by groups of state and individual state. The state groups were populated based on the Socio-demographic Index (SDI) computed by the GBD study, which uses lag distributed income, average years of education for population > 15 years of age, and total fertility rate [9, 32].

To assess the trends in arrests related to domestic violence crimes, we computed the mean number of people arrested under each crime heading by dividing the number of people arrested with the total number of cases. The statistical analysis was done using MS Excel 2016, and maps were created using QGIS [33]. As this analysis used aggregated data available in the public domain, no ethics approval was necessary.

## Results

### Cruelty by husband or his relatives

**Fig. 1:**
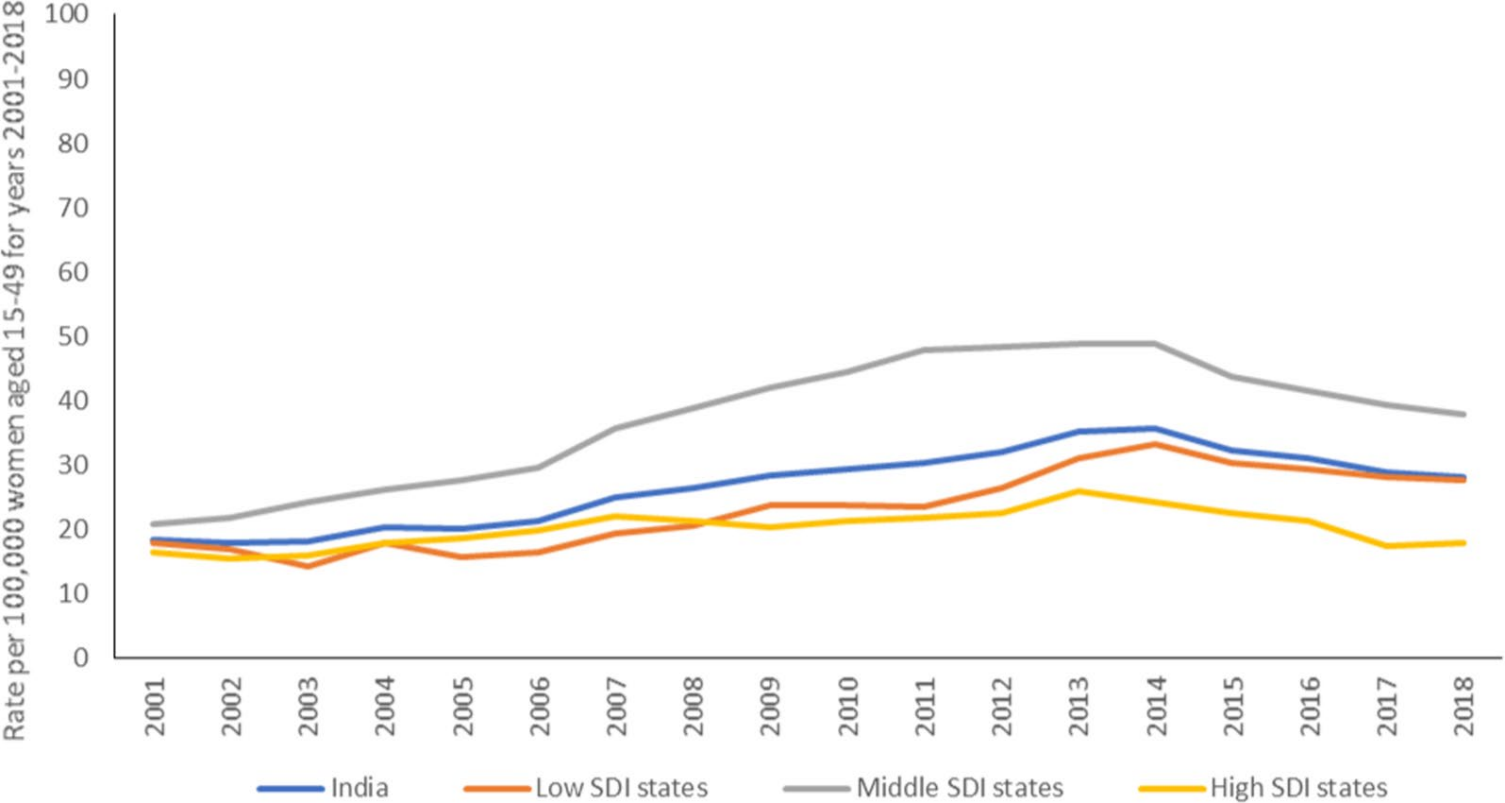
Yearly trend in the rate of cruelty by husband or his relatives per 100,000 women of 15–49 years, 2001–2018. SDI denotes Socio-demographic Index

**Fig. 2:**
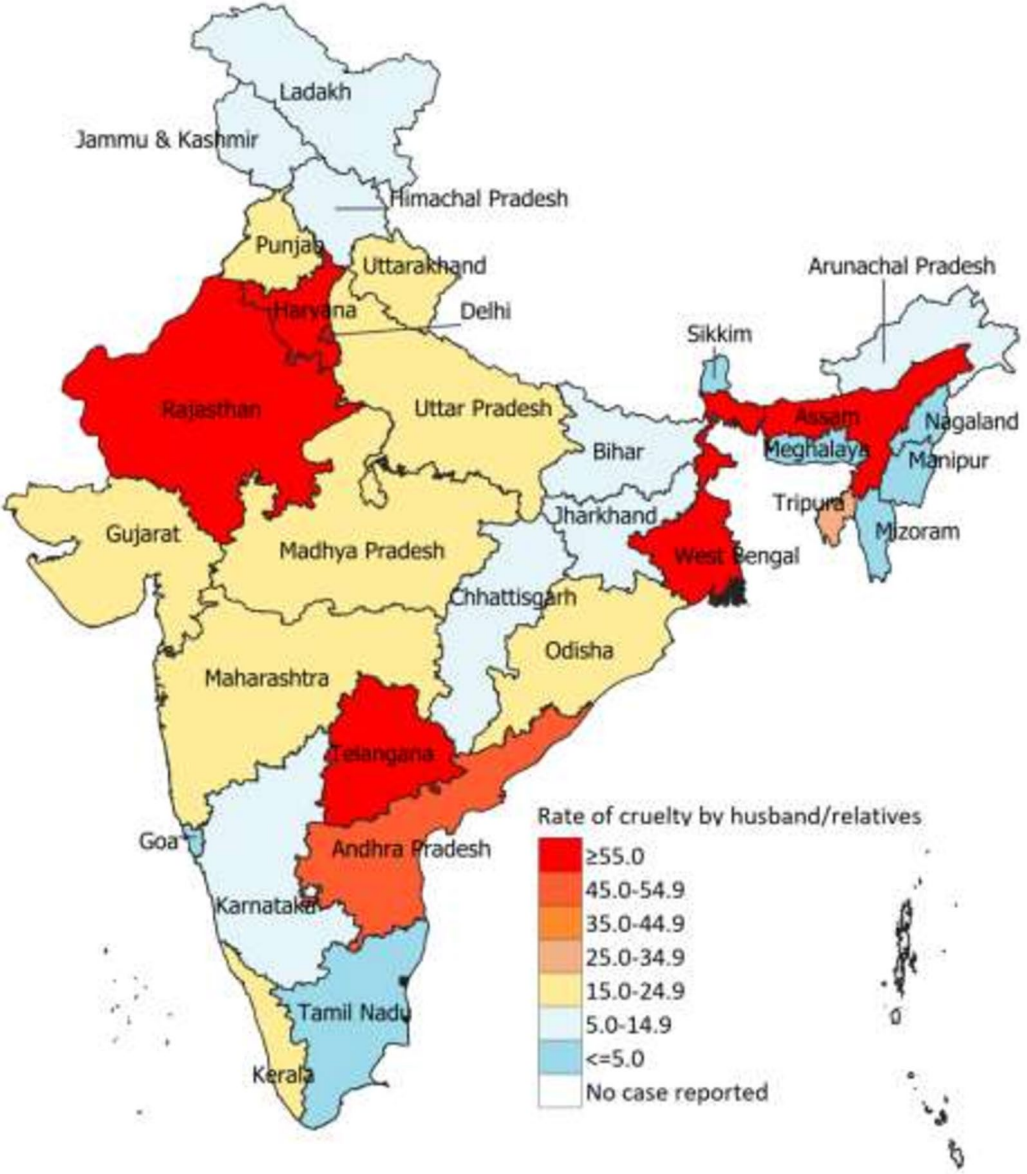
Crime rate for cruelty by husband or his relatives per 100,000 women aged 15–49 years in 2018 in India, by state

A total of 1,548,548 cases were reported under cruelty by husband or his relatives in India from 2001 to 2018, with 554,481 (35.8%) between 2014 and 2018. The reported rate of this crime in India was 18.5 (95% CI 18.3–18.6) in 2001 and 28.3 (95% CI 28.1–28.5) in 2018 per 100,000 women aged 15–49 years, marking a significant increase of 53% (95% CI 51.7–54.3) over this period (Table 1). This rate was 37.9 (95% CI 37.5–38.3) for the middle SDI states as compared with 27.6 (95% CI 27.4–27.8) in the low- and 18.1 (95% CI 17.8–18.4) in the high-SDI states in 2018 (Table 1). This reported crime rate remained higher in the middle SDI states between 2001 and 2018 as compared with the other states, reaching its highest levels between 2011 and 2014 (Fig. 1). Wide variations were seen in the rate for reported cruelty by husband or his relatives in 2018 at the state-level, which ranged from 0.5 (95% CI -0.05 0–1.5) in Sikkim to 113.7 (95% CI 111.6–115.8) in Assam (Table 1 and Fig. 2). The state of Delhi, Assam, West Bengal, Arunachal Pradesh, Meghalaya and Jammu and Kashmir documented > 160% increase in this reported crime rate during 2001–2018 (Table 1). The greatest decline in the rate of this reported crime was seen in Mizoram, 74.3% from 2001 to 2018 (Table 1).

**Table 1:** Rate (with 95% confidence interval) and percent change in rate of domestic violence crimes reported per 100,000 women aged 15–49 by states of India

	Cruelty by Husband or his relatives				Dowry Deaths				Abetment of Suicides of Women^#^		
	Rate			% change 2001–2018	Rate			% change 2001–2018	Rate		% change 2014–2018
	2001	2014	2018		2001	2014	2018		2014	2018	
India	18.5 (18.3–18.7)	35.7 (35.5–35.9)	28.3 (28.1–28.5)	53.0	2.6 (2.5–2.7)	2.5 (2.4–2.5)	2.0 (2.0–2.0)	− 23.1	1.1 (1.1–1.1)	1.4 (1.4–1.4)	27.3
*Low SDI states*	17.9 (17.7–18.1)	33.2 (32.9–33.5)	27.6 (27.4–27.8)	54.2	4.0 (3.9–4.1)	3.8 (3.7–3.9)	3.1 (3.0–3.2)	− 22.5	0.6 (0.6–0.6)	0.7 (0.7–0.8)	16.7
Bihar	7.9 (7.5–8.3)	17.5 (17.0–18.0)	8.6 (8.3–8.9)	8.9	4.4 (4.1–4.7)	5.1 (4.9–5.4)	3.8 (3.6–4.0)	− 13.6	0	0	–
Madhya Pradesh	17.2 (16.5–17.9)	31.8 (31.0–32.5)	18.7 (18.1–19.3)	8.7	4.1 (3.8–4.4)	3.6 (3.3–3.9)	2.5 (2.3–2.7)	− 39.0	2.2 (2.0–2.4)	2.6 (2.4–2.8)	18.2
Jharkhand	7.2 (6.6–7.8)	16.1 (15.3–17.0)	10.6 (10.0–11.2)	47.2	3.2 (2.8–3.6)	3.9 (3.5–4.3)	2.6 (2.3–2.9)	− 18.8	0	0.4 (0.3–0.5)	–
Uttar Pradesh	18.8 (18.4–19.2)	19.2 (18.9–19.6)	23.4 (23.0–23.8)	24.5	5.6 (5.4–5.8)	4.5 (4.4–4.7)	4.0 (3.8–4.2)	− 28.6	0.4 (0.3–0.5)	0.5 (0.4–0.6)	25.0
Rajasthan	40.5 (39.4–41.6)	83.3 (82.0–84.6)	59.1 (58.1–60.1)	45.9	2.8 (2.5–3.1)	2.1 (1.9–2.3)	1.9 (1.7–2.1)	− 32.1	0.7 (0.6–0.8)	0.7 (0.6–0.8)	0.0
Chhattisgarh	15.6 (14.5–16.7)	12.8 (12.0–13.6)	6.1 (5.6–6.6)	− 60.9	1.3 (1.0–1.6)	1.7 (1.4–2.0)	1.0 (0.8–1.2)	− 23.1	1.4 (1.1–1.7)	1.5 (1.2–1.8)	7.1
Odisha	12.8 (12.1–13.5)	25.6 (24.7–26.5)	15.8 (15.1–16.5)	23.4	3.0 (2.7–3.3)	3.6 (3.3–4.0)	3.0 (2.7–3.3)	0.0	0	0.1 (0.0–0.2)	–
Assam	18.0 (17.0–19.0)	105.0 (102.9–107.1)	113.7 (111.6–115.8)	531.7	0.9 (0.7–1.1)	2.1 (1.8–2.3)	1.8 (1.5–2.1)	100	0	0.8 (0.6–1.0)	–
*Middle SDI states*	20.9 (20.6–21.2)	48.9 (48.5–49.3)	37.9 (37.5–38.3)	81.3	1.6 (1.5–1.7)	1.6 (1.6–1.7)	1.2 (1.1–1.3)	− 25.0	1.5 (1.4–1.6)	2.2 (2.1–2.3)	46.7
Andhra Pradesh	45.7 (44.5–46.9)	42.8 (41.7–43.8)	45.3 (44.2–46.4)	− 0.9	3.3 (3.0–3.6)	1.5 (1.3–1.6)	0.9 (0.7–1.1)	− 72.7	1.5 (1.3–1.7)	2.7 (2.4–3.0)	80.0
West Bengal	17.9 (17.3–18.5)	86.2 (85.1–87.3)	61.1 (60.2–62.0)	241.3	1.2 (1.1–1.3)	1.9 (1.7–2.0)	1.6 (1.5–1.7)	33.3	0.4 (0.3–0.5)	1.6 (1.5–1.7)	300.0
Tripura	25.9 (22.5–29.3)	63.9 (59.2–68.7)	26.7 (23.7–29.7)	3.1	1.8 (0.9–2.7)	3.01 (2.0–4.0)	1.6 (0.9–2.3)	− 11.1	0	1.2 (0.6–1.8)	–
Arunachal Pradesh	4.1 (1.7–6.5)	10.0 (7.0–13.1)	12.8 (9.6–16.0)	212.2	0	0.24 (− 0.23 to 0.71)	0	–	0	0.4 (− 0.2 to 1.0)	–
Meghalaya	0.7 (0.0–1.4)	5.0 (3.5–6.5)	2.0 (1.1–2.9)	185.7	0	0.12 (− 0.11 to 0.35)	0.1 (− 0.1 to 0.3)	–	0	0	–
Karnataka	12.1 (11.5–12.7)	16.6 (16.0–17.1)	10.9 (10.4–11.4)	− 9.9	1.5 (1.3–1.7)	1.7 (1.5–1.9)	1.1 (1.0–1.2)	− 26.7	1.2 (1.0–1.4)	1.8 (1.6–2.0)	50.0
Telangana*	–	59.1 (57.7–60.6)	56.3 (54.9–57.7)	–	0	2.7 (2.4–3.0)	1.7 (1.5–1.9)	–	5.8 (5.3–6.3)	4.0 (3.6–4.4)	–31.0
Gujarat	27.1 (26.2–28.0)	34.9 (34.0–35.7)	16.3 (15.7–16.9)	− 39.9	0.5 (0.4–0.6)	0.13 (0.08–0.19)	0.1 (0.1–0.1)	− 80.0	1.0 (0.9–1.1)	2.2 (2.0–2.4)	120.0
Manipur	0.8 (0.1–1.5)	4.6 (3.2–6.0)	1.4 (0.7–2.1)	75.0	0	0.11 (− 0.11 to 0.33)	0	–	0	0.3 (0.0–0.6)	–
Jammu and Kashmir^†^	1.9 (1.4–2.4)	13.8 (12.5–15.0)	8.9 (7.9–9.9)	368.4	0.5 (0.2–0.8)	0.15 (0.02–0.28)	0.2 (0.1–0.3)	− 60.0	0.7 (0.4–1.0)	0.9 (0.6–1.2)	28.6
Haryana	28.6 (27.2–30.0)	49.0 (47.3–50.6)	55.2 (53.5–56.9)	93.0	5.4 (4.8–6.0)	4.1 (3.7–4.6)	2.9 (2.5–3.3)	− 46.3	1.7 (1.4–2.0)	2.7 (2.3–3.1)	58.8
*High SDI states*	16.6 (16.3–16.9)	24.4 (24.0–24.7)	18.1 (17.8–18.4)	9.0	1.3 (1.2–1.4)	0.82 (0.76–0.89)	0.7 (0.6–0.8)	− 46.2	1.5 (1.4–1.6)	1.7 (1.6–1.8)	13.3
Uttarakhand	13.8 (12.2–15.4)	10.4 (9.2–11.5)	19.8 (18.2–21.4)	43.5	2.6 (1.9–3.3)	1.3 (0.9–1.7)	2.0 (1.5–2.5)	− 23.1	0	0.6 (0.3–0.9)	–
Tamil Nadu	4.5 (4.2–4.8)	9.6 (9.2–10.0)	3.6 (3.4–3.8)	− 20.0	1.1 (0.9–1.3)	0.43 (0.35–0.52)	0.2 (0.1–0.3)	− 81.8	0.2 (0.1–0.3)	1.1 (1.0–1.2)	450.0
Mizoram	7.4 (3.8–11.0)	2.7 (0.8–4.6)	1.9 (0.4–3.4)	− 74.3	0	0	0	–	0	0	–
Maharashtra	23.9 (23.3–24.5)	23.9 (23.4–24.4)	20.5 (20.0–21.0)	− 14.2	1.2 (1.1–1.3)	0.87 (0.76–0.97)	0.6 (0.5–0.7)	− 50.0	3.1 (2.9–3.3)	2.6 (2.4–2.8)	–16.1
Punjab	17.7 (16.7–18.7)	21.4 (20.3–22.4)	18.2 (17.3–19.1)	2.8	2.5 (2.1–2.9)	1.1 (0.9–1.4)	0.8 (0.6–1.0)	− 68.0	1.6 (1.3–1.9)	2.2 (1.9–2.5)	37.5
Sikkim	0	2.7 (0.3–5.1)	0.5 (− 0.5 to 1.5)	–	0	0	0.5 (− 0.5 to 1.5)	–	0	0	–
Nagaland	0	0.6 (− 0.1 to 1.2)	0.6 (− 0.1 to 1.3)	–	0	0	0	–	0	0	–
Himachal Pradesh	19.0 (16.9–21.1)	16.3 (14.5–18.1)	9.0 (7.7–10.3)	− 52.6	0.6 (0.2–1.0)	0.05 (− 0.05 to 0.15)	0.2 (0.0–0.4)	− 66.7	3.3 (2.5–4.1)	3.6 (2.8–4.4)	9.1
UTs other than Delhi	7.6 (5.6–9.6)	16.8 (14.2–19.4)	10.6 (8.6–12.6)	39.5	0.5 (0.0–1.0)	0.61 (0.12–1.09)	0.6 (0.1–1.1)	20.0	0.2 (− 0.1 to 0.5)	0.7 (0.2–1.2)	250.0
Kerala	27.2 (26.1–28.3)	51.6 (50.2–53.0)	22.1 (21.1–23.1)	− 18.8	0.3 (0.2–0.4)	0.29 (0.18–0.40)	0.2 (0.1–0.3)	− 33.3	0.4 (0.3–0.5)	0.4 (0.3–0.5)	0.0
Delhi	3.8 (3.2–4.4)	64.2 (62.0–66.5)	65.4 (63.2–67.6)	1621.1	3.1 (2.5–3.7)	3.1 (2.6–3.6)	2.9 (2.4–3.4)	− 6.5	0.6 (0.4–0.8)	0.8 (0.6–1.0)	33.3
Goa	2.8 (1.1–4.5)	8.5 (5.7–11.3)	2.2 (0.8–3.6)	− 21.4	0.5 (− 0.2 to 1.2)	0	0	− 100.0	0	0.5 (− 0.2 to 1.2)	–

	Protection of Women From Domestic Violence Act, 2005^#^			All Domestic Violence Related Crimes							
	Rate		% change 2014–2018	Rate		% change 2014–2018					
	2014	2018		2014	2018						
India	0.1 (0.1–0.1)	0.2 (0.2–0.2)	No change	39.4 (39.2–39.6)	31.8 (31.6–32.0)	− 19.3					
*Low SDI states*	0.2 (0.2–0.2)	0.2 (0.2–0.2)	No change	37.8 (37.5–38.1)	31.6 (31.3–31.9)	− 16.4					
Bihar	0.4 (0.3–0.5)	− 100	− 100	23.0 (22.4–23.6)	12.4 (12.0–12.8)	− 46.1					
Madhya Pradesh	0.3 (0.2–0.4)	1.2 (1.1–1.3)	300	37.9 (37.1–38.7)	25.0 (24.3–25.7)	− 34.0					
Jharkhand	0.1 (0.0–0.2)	0.8 (0.6–1.0)	700	20.1 (19.2–21.0)	14.3 (13.6–15.0)	− 28.9					
Uttar Pradesh	0.1 (0.1–0.1)	0	No change	24.3 (23.9–24.7)	27.9 (27.5–28.3)	14.8					
Rajasthan	0.1 (0.1–0.1)	0	No change	86.3 (85.0–87.6)	61.8 (60.7–62.9)	− 28.4					
Chhattisgarh	0	0	–	15.9 (15.0–16.8)	8.5 (7.9–9.1)	− 46.5					
Odisha	0	0	–	29.2 (28.2–30.2)	18.9 (18.1–19.7)	− 35.3					
Assam	0	0	–	107.1 (105.0–109.2)	116.4 (114.3–118.5)	8.7					
*Middle SDI states*	0	0	–	52.0 (51.6–52.4)	41.2 (40.8–41.6)	− 20.8					
Andhra Pradesh	0	0	–	45.7 (44.6–46.8)	48.9 (47.8–50.0)	7.0					
West Bengal	0	0	–	88.5 (87.4–89.6)	64.3 (63.4–65.2)	− 27.3					
Tripura	0	0	–	66.9 (62.1–71.7)	29.5 (26.3–32.7)	− 55.9					
Arunachal Pradesh	0	0.4 (− 0.2 to 1.0)	–	10.3 (7.2–13.4)	13.6 (10.3–16.9)	32.0					
Meghalaya	0	0	–	5.1 (3.6–6.6)	2.1 (1.2–3.0)	− 58.8					
Karnataka	0	0	–	19.5 (18.9–20.1)	13.8 (13.3–14.3)	− 29.2					
Telangana*	0	0	–	67.7 (66.1–69.3)	61.9 (60.4–63.4)	− 8.6					
Gujarat	0	0	–	36.0 (35.1–36.9)	18.5 (17.9–19.1)	− 48.6					
Manipur	0	0	–	4.7 (3.3–6.1)	1.8 (1.0–2.6)	− 61.7					
Jammu and Kashmir^†^	0	0	–	14.6 (13.3–15.9)	10.0 (9.0–11.0)	− 31.5					
Haryana	0.1 (0.0–0.2)	0	No change	54.8 (53.1–56.5)	60.8 (59.0–62.6)	10.9					
*High SDI states*	0.2 (0.2–0.2)	0.2 (0.2–0.2)	0	26.9 (26.5–27.3)	20.7 (20.4–21.0)	− 23.0					
Uttarakhand	0	0	–	11.7 (10.5–12.9)	22.4 (20.7–24.1)	91.5					
Tamil Nadu	0	0	–	10.3 (9.9–10.7)	4.9 (4.6–5.2)	− 52.4					
Mizoram	0	0.3 (− 0.3 to 0.9)	–	2.7 (0.8–4.6)	2.2 (0.6–3.8)	− 18.5					
Maharashtra	0	0	–	27.8 (27.2–28.4)	23.8 (23.3–24.3)	− 14.4					
Punjab	0	0	–	24.1 (23.0–25.2)	21.2 (20.2–22.2)	− 12.0					
Sikkim	0	0	–	2.7 (0.3–5.1)	1.1 (− 0.4 to 2.6)	− 59.3					
Nagaland	0	0	–	0.6 (− 0.1 to 1.3)	0.6 (− 0.1 to 1.3)	0.0					
Himachal Pradesh	0.3 (0.1–0.5)	0.4 (0.1–0.7)	33	19.9 (17.9–21.9)	13.2 (11.6–14.8)	− 33.7					
UTs other than Delhi	0	0	–	17.6 (15.0–20.2)	11.9 (9.8–14.0)	− 32.4					
Kerala	1.5 (1.3–1.7)	1.9 (1.6–2.2)	27	53.8 (52.3–55.3)	24.6 (23.6–25.6)	− 54.3					
Delhi	0.1 (0.0–0.2)	0	No change	68.0 (65.7–70.3)	69.3 (67.0–71.6)	1.9					
Goa	0.5 (− 0.2 to 1.2)	0	No change	8.9 (6.0–11.8)	2.7 (1.1–4.3)	− 69.7					

SDI denotes Socio-demographic Index

*Telangana state was formed in 2014

^#^Data available from 2014 onwards

Interestingly, the 53% increase in this reported crime rate between 2001 and 2018 for India was accounted for by increased rates for only a few states, and the rate remained stagnant in most states (Additional file 2: Fig. S1, Additional file 3: Fig. S2, Additional file 4: Fig. S3). Only the states of Assam and Rajasthan among the low SDI states (Additional file 2: Fig. S1), Andhra Pradesh and Tripura among the middle SDI states (Additional file 3: Fig. S2), and Kerala and Delhi among the high SDI states (Additional file 4: Fig. S3) showed increased reporting of this crime over the study period. The mean number of persons arrested under this crime in India decreased from 2.2 in 2001 to 1.1 in 2018, and the numbers were similar across the state SDI groups (Additional file 5: Fig. S4).

### Dowry deaths

**Fig. 3:**
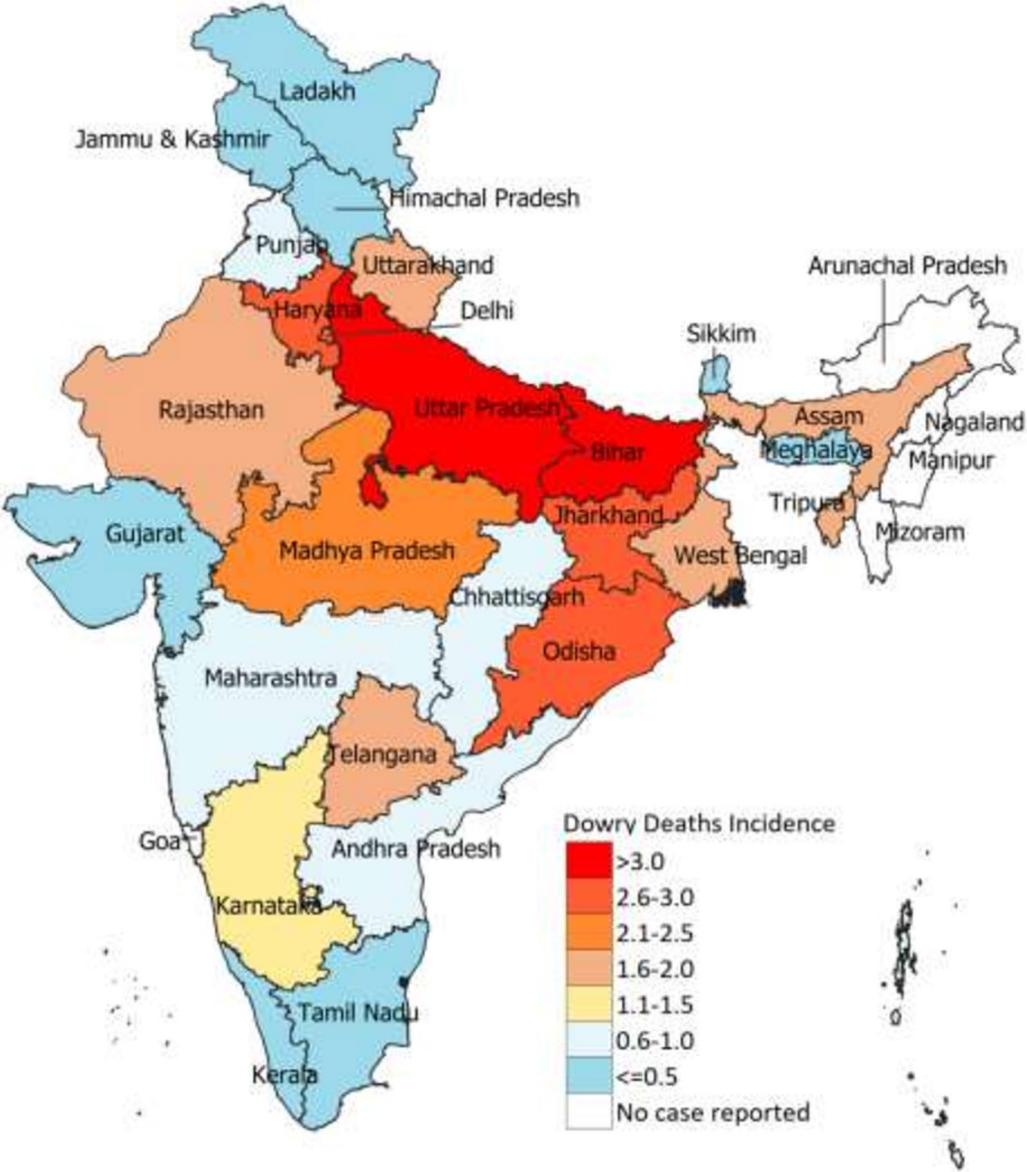
Rate of dowry deaths per 100,000 women aged 15–49 years in 2018 in India, by state

A total of 137,627 crimes were reported as dowry deaths between 2001 and 2018, with 38,342 (27.9%) cases between 2014 and 2018. The rate of this reported crime in India was 2.0 (95% CI 2.5–2.7) in 2018 per 100,000 women aged 15–49 years (Table 1). This rate in 2018 was 3.1 (95% CI 3.0–3.2) in the low-SDI states as compared to 1.2 (95% CI 1.1–1) in the middle- and 0.7 (95% CI 0.60–0.8) in the high-SDI states, and this trend was seen throughout the period studied (Table 1). At the state level in 2018, this rate ranged from 0.11 (95% CI 0–0.32) in Meghalaya to 4.0 (95% CI 3.8–4.2) in Uttar Pradesh; no cases were reported in Arunachal Pradesh, Manipur, Mizoram or Nagaland (Table 1 and Fig. 3). The largest decline in this rate was seen in the states of Tamil Nadu and Gujarat over the study period (Table 1).

The mean number of persons arrested for dowry deaths in India declined from 3 in 2001 to 2.3 in 2018. In 2001, this mean was markedly higher in the high-SDI states (4.9) than the low- (2.7) and middle- (2.6) SDI states. However, by 2015 this rate was higher in the low-SDI states (2.9) than high- (2.2) and middle- (1.8) SDI states (Additional file 5: Fig. S4).

### Abetment of suicide of women

Data under this crime head was available from 2014 to 2018, during which 22,579 cases were reported. The average rate of this crime was 1.27 (95% CI 1.25–1.29) per 100,000 women aged 15–49 years over this period. Overall, relatively higher rates were recorded in middle-SDI states (2.2; 95% CI 2.1–2.3), followed by high- (1.7; 95% CI 1.6–1.8) and low- (0.73; 95% CI 0.69–0.77) SDI states (Table 1). Notably, the middle- and high-SDI groups recorded a similar rate in 2014, after which the middle-SDI states recorded a steady increase in rate until 2017, while the high-SDI states saw an initial dip in 2015 and then an increase till 2017. The rate in the low-SDI states remained low throughout this period (Table 1).

**Fig. 4:**
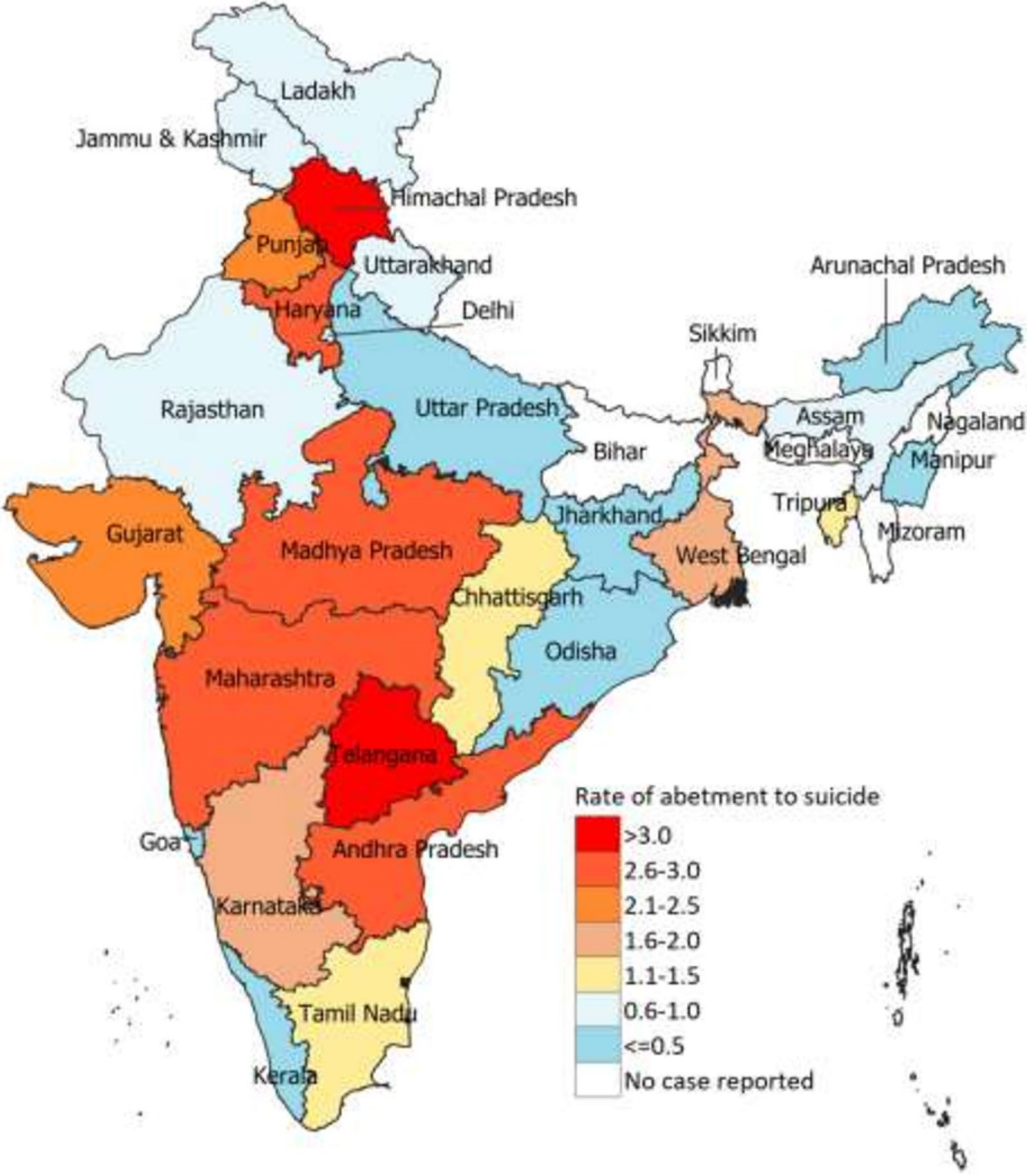
Rate of abetment of suicide of women per 100,000 women aged 15–49 years in 2018 in India, by state

At the state-level in 2018, this rate ranged from 0.07 (95% CI 0.02 to 0.12) in Odisha to 4.0 (95% CI 3.6–4.4) in Telangana; no cases were reported in Bihar, Meghalaya, Mizoram, Sikkim and Nagaland (Table 1 and Fig. 4). While some states did not record any case, other states recorded significant changes between the 2014 and 2018. This rate in Tamil Nadu increased by 450% from 2014 to 2018, and West Bengal and Gujarat recorded an increase of over 100%, while this rate declined the most in Telangana, by 31% (Table 1). The mean number of persons arrested for this crime in India recorded a small increase from 1.4 in 2014 to 1.7 in 2018, and was similar across the state SDI groups (Additional file 5: Fig. S4).

### PWDVA, 2005

A total of 2,519 cases were reported under PWDVA between 2014 and 2018, with an average crime rate of 0.14 (95% CI 0.13–0.15) per 100,000 women aged 15–49 years during this period (Table 1). Majority of the states did not report any case under this Act (Table 1). The mean number of persons arrested in India for this crime decreased from 1.6 in 2014 to 1 in 2018 (Additional file 5: Fig. S4).

### Status of the legal cases

A total of 658,418 cases were sent for trial in India in 2018, of which trial was completed in only 44,648 (6.8%) cases. Among the cases in which trial was completed, the offender(s) was convicted in only 6,921 (15.5%) cases.

## Discussion

In India between 2001 and 2018, the majority of domestic violence cases were filed under ‘cruelty by husband or his relatives’, with the reported rate of this crime increasing by 53% over the 18 years. However, it is important to note that only some states recorded change in the reported rate with the almost stagnant reported rate of domestic violence in many states over time. Significant heterogeneity was seen in the pattern of the four types of crimes at the state-level. Overall, the mean persons arrested decreased irrespective of the crime during the period studied, and less than 7% of the filed cases had completed legal trial in 2018. We discuss the gaps identified in the reported data which unless addressed have major implications in the facilitating action to reduce domestic violence against women in India.

The rate of reported crime under all the considered categories excluding dowry deaths in 2018 in India in the NCRB was close to the 33% self-reported domestic violence reported by women in the national survey in 2015–16 [3], though there is an indication that the prevalence of domestic violence could be as high as 41% in India [4]. The NCRB data provides passive surveillance with the source being the FIR filed by family/kin/community member with the police for a crime, and hence is dependent on the reporting from the community, which is known to be selective as women report less to the police for domestic violence due to various reasons including lack of social support, shame, and stigma [34–37]. These differences could account for differential rates of domestic violence between the police records and self-reporting of domestic violence in the surveys [3, 4]. Recently, it is also shown that how women are asked about domestic violence in surveys can also result in different estimates [38]. Furthermore, the Principal Offence Rule followed by NCRB "hides" many cases of domestic violence as according to this Rule, each criminal incident is recorded as one crime. If many offences are registered in a single case, only the most heinous crime—one that attracts maximum punishment—is considered as counting unit [39, 40]. For example, an incident involving dowry death and cruelty by husband or relative will be reported in NCRB as dowry death as it warrants the maximum punishment, thereby, underreporting the number of cases with cruelty by husband or relative.

### Cruelty by husband or his relatives

The cases under cruelty by husband or his relatives accounted for the majority of reported cases, and the rate of this reporting was comparatively higher in the middle-SDI states over the years studied. Previous research using field notes from cases reported to police indicate that victims are often in an environment that condones violence through active encouragement or tacit approval by the husband’s family members; and that many women lack social support as they experience violence from multiple perpetrators at home [34, 41]. It is plausible that this rate is higher in the middle-SDI states because material wealth is highly prized among the Indian middle class, and dowry is seen as an easy path to greater wealth and social status [12]. A higher dowry demand, and a greater dissatisfaction from inability to meet these demands could possibly result in more domestic violence in these states [12, 42, 43]. Another possible factor in these states could be that the increasing female literacy in these states may be perceived as a threat to the prevalent power structures, prompting violence against women as a means to reinstate control [12, 44–47].

### Abetment of suicide of women

The middle-SDI states also had a higher rate of reported cases under abetment to suicide. The link between abuse and suicidal behaviour is well established, with research indicating that three out of ten women who undergo domestic violence are likely to attempt suicide [5]. Furthermore, a significantly higher suicide death rate is reported in Indian women than their global counterparts [9], and housewives account for the majority of these suicide deaths [10]. Wide state-level variations in the suicide death rate for women are also reported [9], and the relationship between the prevalence of domestic violence and suicide death rate needs to be explored further.

### Dowry deaths

In contrast to the increased reporting of cases of cruelty over time, the rate of dowry death cases decreased from 2001 to 2018, with the low-SDI states recording the highest rate of dowry deaths. The dip in these cases may have resulted from the 2010 judgment requiring prior harassment of the victim associated with a dowry shortfall which made it harder to register a dowry death but presumably also harder to prove beyond a reasonable doubt that it was a dowry death, and not in fact.[48] Furthermore, qualitative research has shown that the families of dowry death cases deter from accusing the husband or his family due to fear of issues with up-bringing of the children of their daughter [47]. Also dowry deaths or related suicide deaths are less likely to be reported by the natal family, who fear social stigma and negative impact on marriages of their other daughters [42, 49]. In this context, it is not easy to interpret the decreased number of cases of dowry deaths in India as actual fewer dowry deaths, for which more evidence is needed.

### PWDVA, 2005

Very few cases were filed under PWDVA with the middle-SDI states reporting no cases during the period studied. While PWDVA defines domestic violence to include coercive behaviour as well as physical, sexual, emotional and economic abuse [11], in actuality only extreme forms of physical violence with evidence of injury are seen to evoke a legal response [12]. Interviews with victims indicate that unless they were able to offer a dowry claim or show evidence of grave physical violence, the police were either reluctant to file an FIR or offer PWDVA as a legal recourse to them [12]. It is also documented that the police, acting as social brokers, attempt to fit the reported domestic violence cases into ‘normal constructs’ frequently focusing on dowry harassment despite the broadened scope of the law as a recourse for domestic violence beyond dowry harassment [5]. Thus, data under this crime heading is unlikely to reflect the true picture of domestic violence against women in India.

### Status of the legal cases

The poor response of formal system to domestic violence is also reflected in the legal recourse as only 6.8% of the cases filed completed trials in 2018, with the majority of accused being acquitted. This bleak state of waiting, extended trials and low conviction is known to further discourage women from reporting [50]. The legal process is also influenced by the patriarchy driven attitudes of the police and people in the legal systems [44], and their unwillingness to act on domestic violence cases which they view as “private matter,”[13, 44] such that many cases are not investigated, or dropped due to delay in filing [5]. In other cases, the investigation is based on the statement of the husband or relatives rather than fingerprints [13], with the perpetrator of violence not even recorded in over 90% of the cases [5]. Notably, little empirical research is available on the perceptions of abusive husbands and families on domestic violence that can facilitate intervention programs for abusive husbands [34].

### Limitations and way forward

There are limitations to the data presented and the interpretation. The NCRB data depend on the availability and quality of data recorded by the police at the local level, which is known to have varied quality [9, 10, 18]. The findings have to be interpreted within in this limitation as it is not possible for us to comment on the extent of underreporting of data or the pattern of underreporting by type of crime, year or state. The heterogeneity in the NCRB data at the state level highlighted by the noisy trends or stagnant trend for certain states do not allow for a meaningful interpretation, and calls for a robust assessment of the reporting practices by the police and judiciary at state level to identify the gaps for inadequate documentation and underreporting that can facilitate appropriate corrective measures to improve data quality [18]. We assumed the age group of affected women to be 15–49 years. Though majority of the cases are likely to be in this age group given the other available information, the unavailability of age of women affected by the type of crime, year and state restricts understanding of the target women for prevention and action. Currently, the data are available in heavily tabulated fixed formats that limit the extent of disaggregated analysis. Because of non-availability of data on number of victims for some years, we assumed the ratio of the number of cases to victims based on the available data for other years. More informative analyses may also be possible if the NCRB reports allow for anonymized individual level data to be available in the public domain, including repeat reports of domestic violence by individual women.

Despite NCRB being a passive surveillance source, efforts can be made to improve the quality of information collected by the police during their routine tasks to improve utilisation of these data for planning action. The World Health Organization injury surveillance guidelines could provide practical advice on collecting systematic data on domestic violence, which can be more comparable over time and location [51]. Training and sensitisation of the police to address gender violence should also include standardisations in capturing of the data and the quality of data captured.

Disasters, natural or otherwise, disproportionately impact women and girls with some evidence suggesting that violence against women increases in disaster settings, however, there is a lack of rigorously designed and good quality studies that are needed to inform evidence-based policies and safeguard women and girls during and after disasters [52]. There has also been suggestion of an increase in domestic violence against women during the Covid-19 pandemic, globally [53] and in India [54, 55]. In this context, the urgency to address the gaps highlighted in the NCRB data is even more for India to protect its women against domestic violence.

## Conclusions

India needs to address the gaps in the administrative data to effectively respond to the SDG target five to eliminate all forms of violence against women. This longitudual analysis of the reported cases of domestic violence of nearly 20 years across the Indian states has highlighted the under-reporting and almost stagnant data, which hinders formulating of well-informed public health intervention strategies to reduce domestic violence in India.

## Supplementary Information

**Additional file 1**. Definitions of crime headings considered under domestic violence. IPC denotes Indian Penal Code.

**Additional file 2**. Crime rate for cruelty by husband or his relatives per 100,000 women aged 15-49 years in the Indian states categorised as having low Socio-demographic Index, 2001-18.

**Additional file 3.** Crime rate for cruelty by husband or his relatives per 100,000 women aged 15-49 years in the Indian states categorised as having middle Socio-demographic Index, 2001-18. Telangana state not shown as it was formed in 2014.

**Additional file 4.** Crime rate for cruelty by husband or his relatives per 100,000 women aged 15-49 years in the Indian states categorised as having high Socio-demographic Index, 2001-18.

**Additional file 5.** Mean numbers of persons arrested under cruelty by husband or his relatives and dowry deaths for the years 2001-2018, and under abetment of suicide of women and PWDVA for the years 2014-2018. SDI denotes Socio-demographic Index. The state wise data for mean number of persons arrested was available until 2015 only.

## Data Availability

The domestic violence related data used in these analyses are available at NCRB website (https://ncrb.gov.in) and from the authors on request. The GBD population data used in these analyses are available at GBD Results Tool | GHDx (healthdata.org).

## Abbreviations

CI: Confidence interval
FIR: First information report
GBD: Global burden of disease
IPC: Indian penal code
NCRB: National Crimes Records Bureau
NFHS: National Family Health Survey
PWDVA: Protection of women from domestic violence act
QGIS: Quantum geographic information system
SDG: Sustainable development goal
SDI: Socio-demographic index
WHO: World Health Organization
