# Supplementary material for: Domestic violence in Indian women: lessons from nearly 20 years of surveillance
Source: BMC Womens Health. 2022 Apr 21;22:128. doi: 10.1186/s12905-022-01703-3 (PMC9023044; doi:10.1186/s12905-022-01703-3)
Supplement: Supplementary file 1 — Additional file 1. Definitions of crime headings considered under domestic violence. IPC denotes Indian Penal Code. [file 12905_2022_1703_MOESM1_ESM.docx]

Additional Table 1. Definitions of crime headings considered under domestic violence. IPC denotes Indian Penal Code.

| **Crime heading** | **IPC section** | **IPC definition** |
| --- | --- | --- |
| Cruelty by husband or his relatives | 498A | Whoever, being the husband or the relative of the husband of a woman, subjects such woman to cruelty shall be punished with imprisonment for a term which may extend to three years and shall also be liable to fine.  For the purposes of this section, “cruelty” means—  (a) any wilful conduct which is of such a nature as is likely to drive the woman to commit suicide or to cause grave injury or danger to life, limb or health (whether mental or physical) of the woman;  or  (b) harassment of the woman where such harassment is with a view to coercing her or any person related to her to meet any unlawful demand for any property or valuable security or is on account of failure by her or any person related to her to meet such demand. |
| Dowry death | 304B | Where the death of a woman is caused by any burns or bodily injury or  occurs otherwise than under normal circumstances within seven years of her marriage and it is shown that soon before her death she was subjected to cruelty or harassment by her husband or any relative of her husband for, or in connection with, any demand for dowry, such death shall be called “dowry death”, and such husband or relative shall be deemed to have caused her death  Whoever commits dowry death shall be punished with imprisonment for a term which shall not be less than seven years but which may extend to imprisonment for life.  Explanation-For the purposes of this sub-section, “dowry” shall have the same meaning as in section 2 of the Dowry Prohibition Act, 1961 (28 of 1961). |
| Abetment to suicide | 306 | If any person commits suicide, whoever abets the commission of such  suicide, shall be punished with imprisonment of either description for a term which may extend to ten years, and shall also be liable to fine. |
| Protection of women against domestic violence Act* | Act, 2005 by the legislative department | An Act to provide for more effective protection of the rights of women guaranteed under the Constitution who are victims of violence of any kind occurring within the family and formatters connected therewith or incidental thereto. |

*Crime is registered under this Act; detailed definition available at <https://legislative.gov.in/actsofparliamentfromtheyear/protection-women-domestic-violence-act-2005>
