# Supplementary figures and images for: Domestic violence in Indian women: lessons from nearly 20 years of surveillance
Source: BMC Womens Health. 2022 Apr 21;22:128. doi: 10.1186/s12905-022-01703-3 (PMC9023044; doi:10.1186/s12905-022-01703-3)

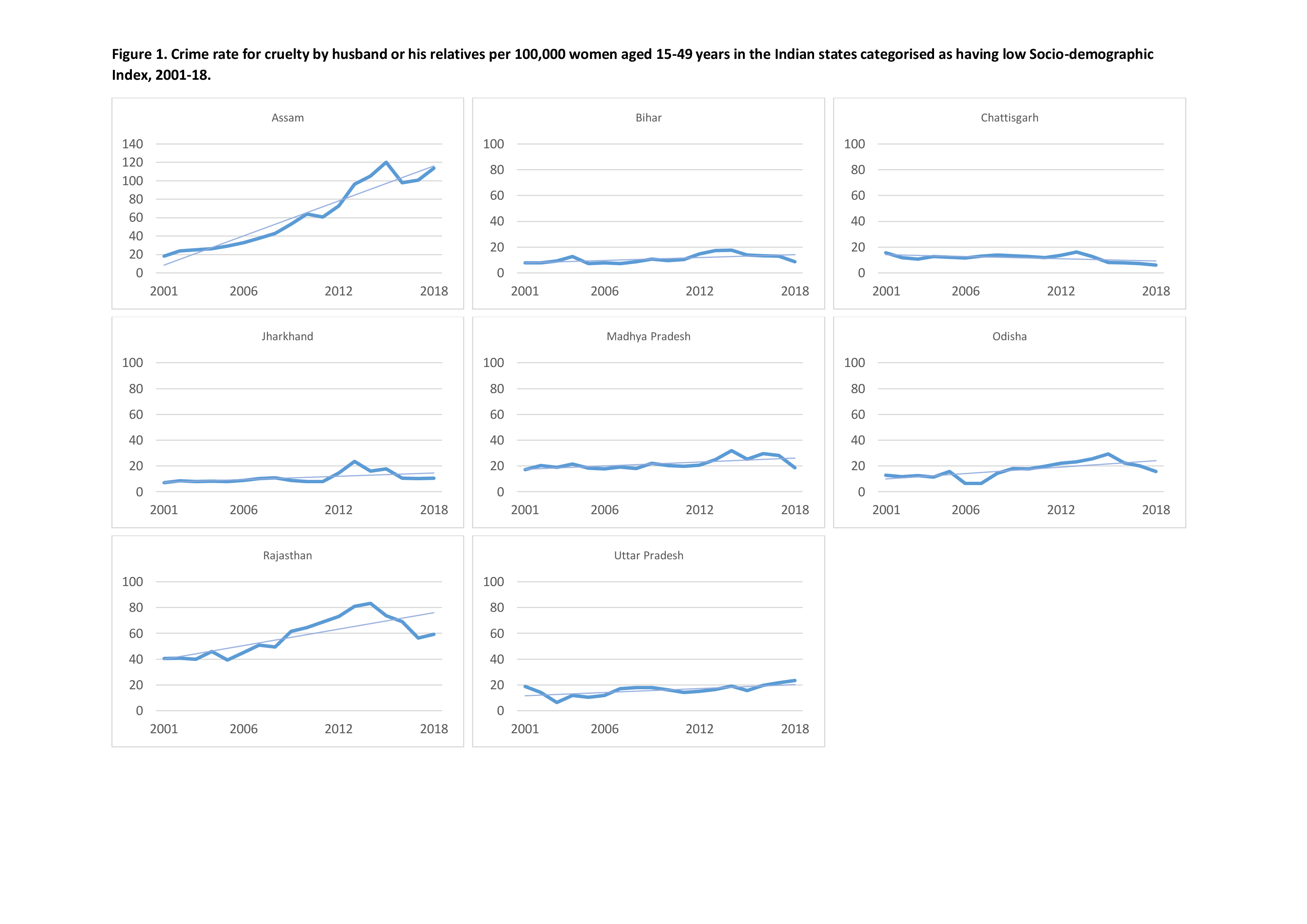

Supplement: Supplementary file 2 — Additional file 2. Crime rate for cruelty by husband or his relatives per 100,000 women aged 15-49 years in the Indian states categorised as having low Socio-demographic Index, 2001-18. [file 12905_2022_1703_MOESM2_ESM.tiff]

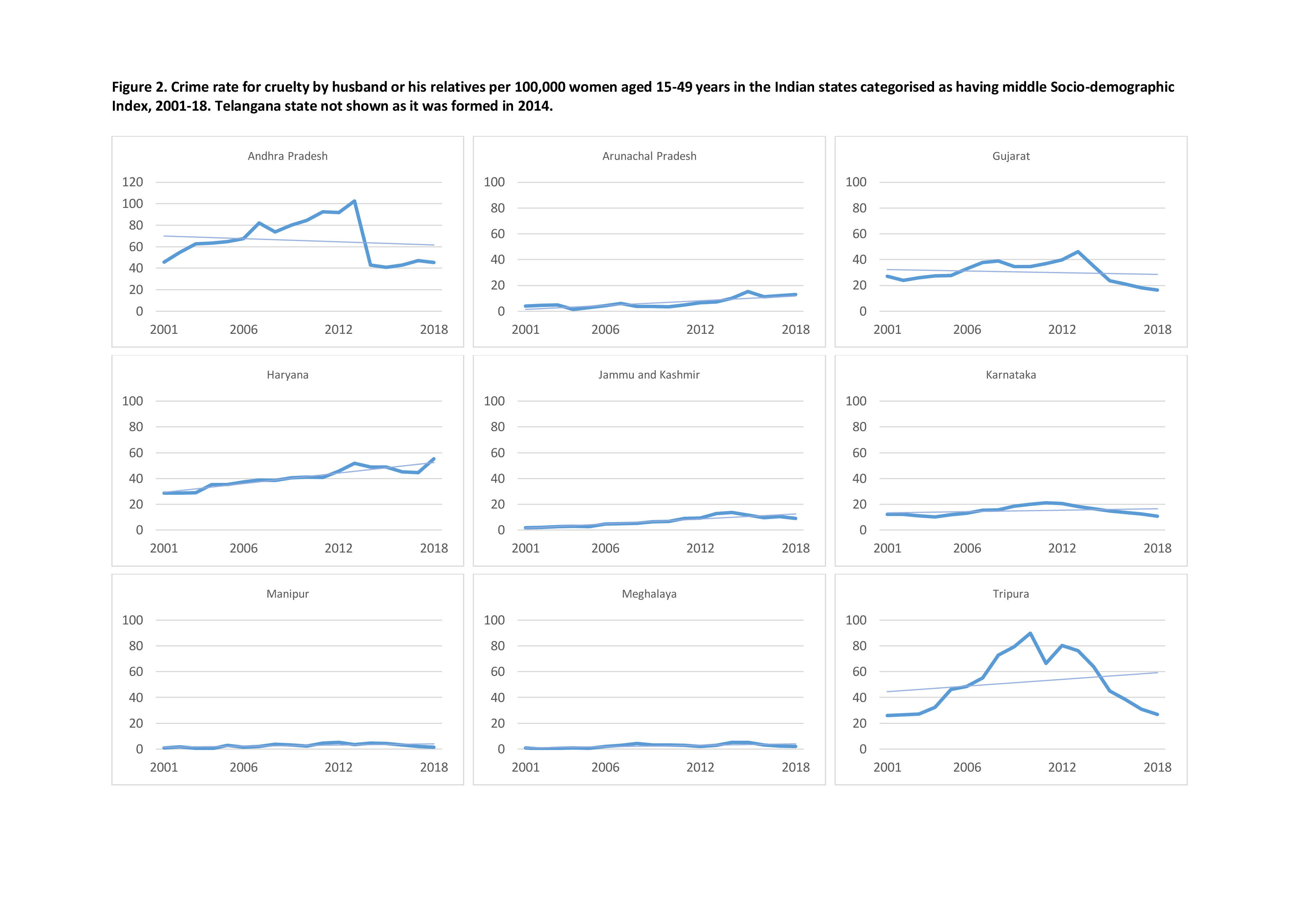

Supplement: Supplementary file 3 — Additional file 3. Crime rate for cruelty by husband or his relatives per 100,000 women aged 15-49 years in the Indian states categorised as having middle Socio-demographic Index, 2001-18. Telangana state not shown as it was formed in 2014. [file 12905_2022_1703_MOESM3_ESM.tiff]

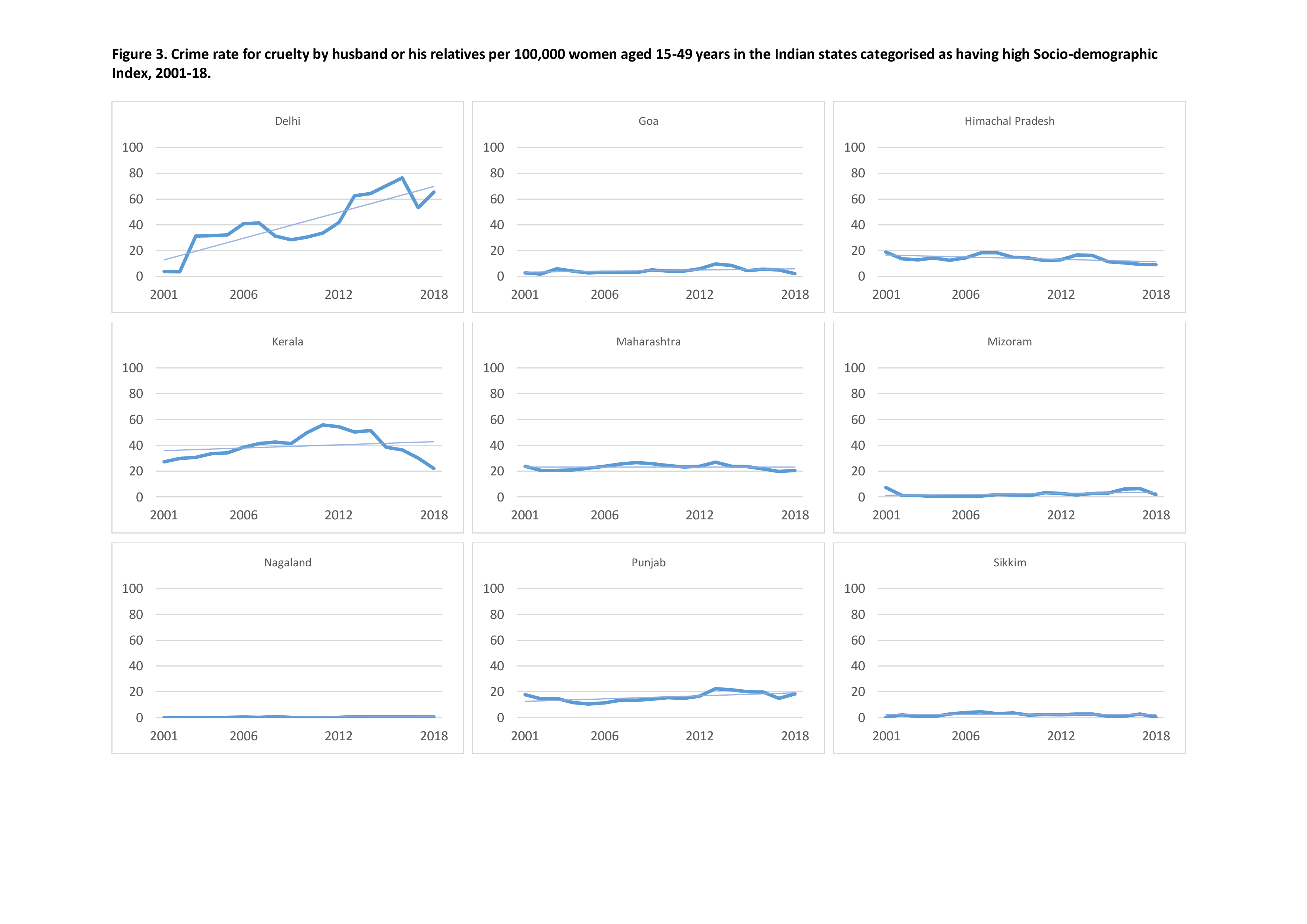

Supplement: Supplementary file 4 — Additional file 4. Crime rate for cruelty by husband or his relatives per 100,000 women aged 15-49 years in the Indian states categorised as having high Socio-demographic Index, 2001-18. [file 12905_2022_1703_MOESM4_ESM.tiff]

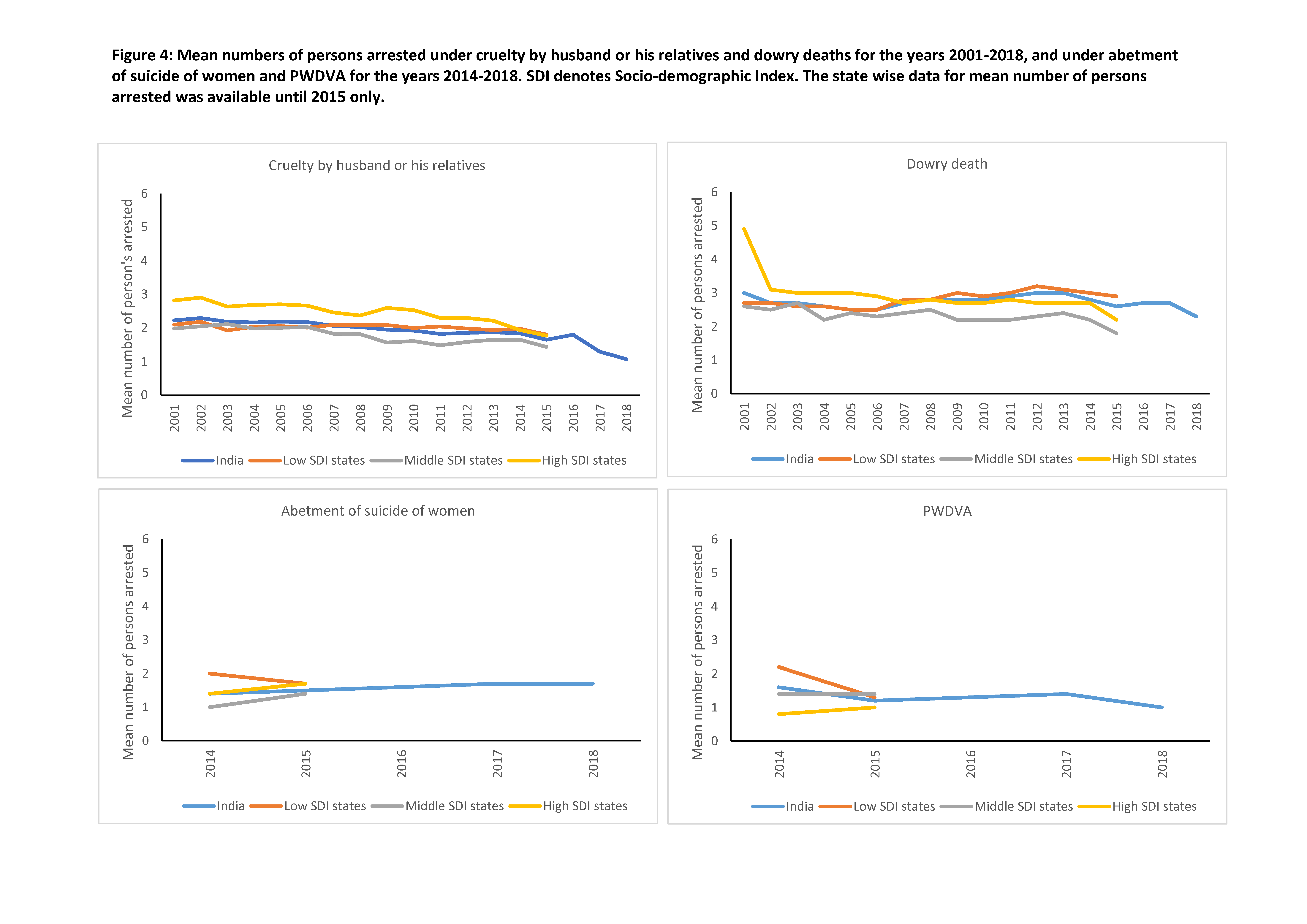

Supplement: Supplementary file 5 — Additional file 5. Mean numbers of persons arrested under cruelty by husband or his relatives and dowry deaths for the years 2001-2018, and under abetment of suicide of women and PWDVA for the years 2014-2018. SDI denotes Socio-demographic Index. The state wise data for mean number of persons arrested was available until 2015 only. [file 12905_2022_1703_MOESM5_ESM.tiff]
